# Editorial: Global excellence in pharmacology of infectious diseases: Australia and Asia

**DOI:** 10.3389/fphar.2023.1243284

**Published:** 2023-07-13

**Authors:** Kwang-sun Kim

**Affiliations:** Department of Chemistry, Chemistry Institute for Functional Materials, Pusan National University, Busan, Republic of Korea

**Keywords:** infectious disease, antibiotic resistance, malaria, bacteriophage, hybrid antibiotics, heparin

**FIGURE 1:**
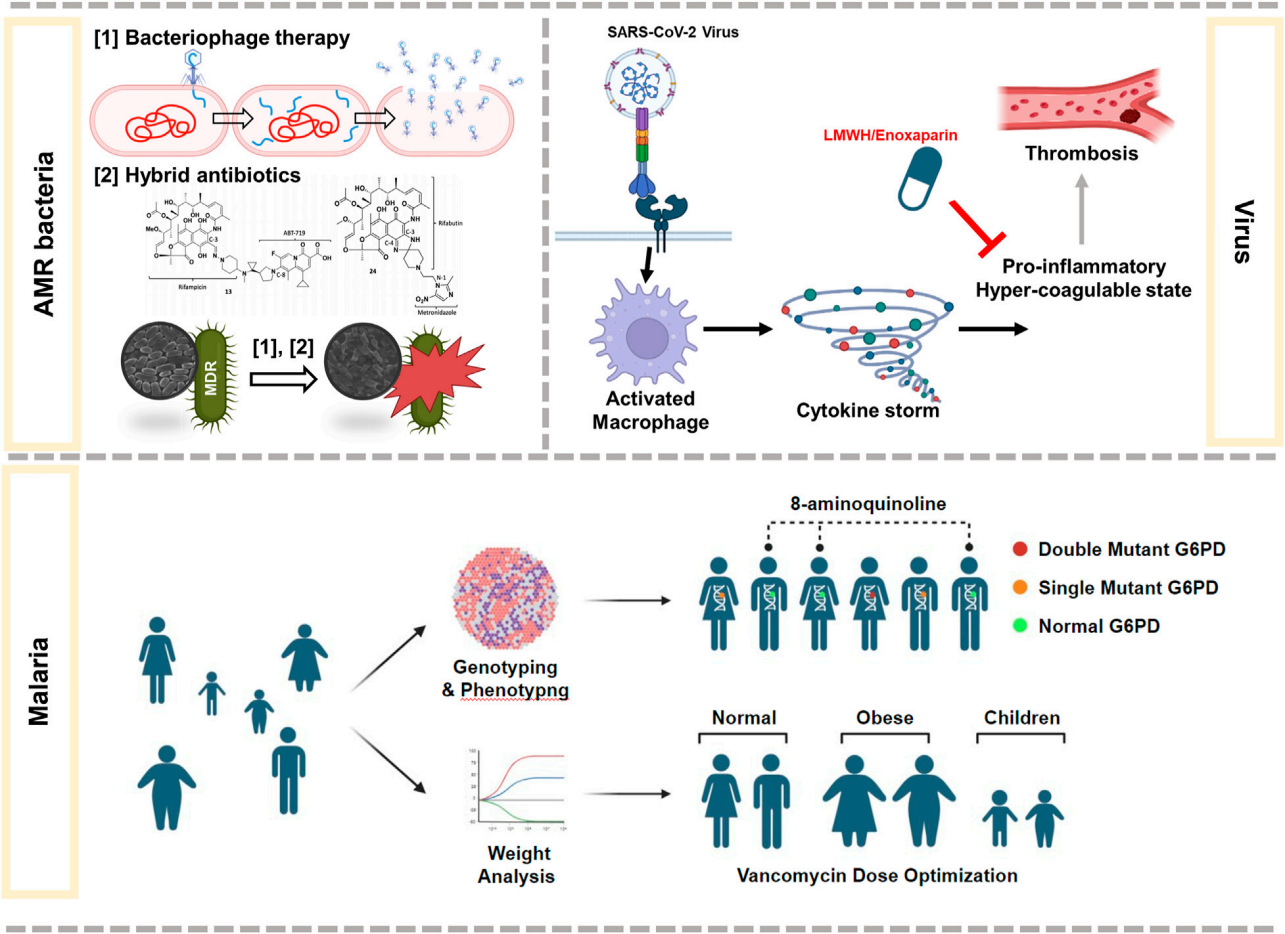
Summary of some of the latest research on therapies against infectious diseases caused by AMR bacteria, virus, or malaria.

Infectious diseases are the most significant threat worldwide, causing death by pathogenic microorganisms, such as bacteria, viruses, fungi, or parasites, that may pass from person to person, either directly or indirectly. The COVID-19 pandemic has shown that infectious disease outbreaks can result in high levels of mortality, significant disability burdens, and disastrous repercussions. In this study, I highlight the latest research on current therapies for health-threatening diseases caused by bacteria, parasites, or viruses, as well as their challenges (Figure 1).

Malaria is a life-threatening disease that spreads to humans through mosquitoes. In 2021, nearly half of the world’s population was at risk of malaria, with an estimated 619, 000 deaths (WHO, 2022). *Plasmodium vivax* is the dominant malaria parasite in humans in Asia and the Asia-Pacific region (Howes et al., 2016). As a therapeutic option, 8-aminoquinolines (primaquine and tafenoquine) have been used to prevent malaria; however, safe and effective anti-relapse therapy against people with glucose-6-phosphate dehydrogenase (G6PD) deficiency is challenging. Sudsumrit et al. assessed the prevalence of hemolytic toxicity in individuals in the malaria-endemic area of Thailand and suggested potential eligible individuals for radical treatment with 8-aminoquinolines. Initially, the authors identified 12 mutations in by G6PD deficiency using quantitative multiplexed high-resolution melting, biochemical, and structural stability assays in 1,125 Thai individuals. Based on these results, the authors demonstrated that individuals with double G6PD mutations are more likely to suffer from hemolysis than those with single G6PD mutations.

The ineffectiveness of current antibiotics and lack of new antibiotics in the pipeline have accelerated the spread of antimicrobial resistant (AMR) bacteria (Pulingam et al., 2022). The World Health Organization predicts that the number of people suffering from AMR infections will increase to 10 million by 2050 if improper and excessive use of antibiotics continue (WHO, 2014; de Kraker et al., 2016; Pulingam et al., 2022). Furthermore, antibiotics can kill beneficial bacteria indiscriminately. Therefore, immediate action is required to combat AMRs using novel and specific strategies to target AMR bacteria. Elrggal et al. conducted a comprehensive study on vancomycin dosage optimization in patients with obesity. Individuals with obesity account for approximately 50% of all acute bacterial skin and skin structure infections in the United States (McGinnis et al., 2018). However, the US Food and Drug Administration (FDA) does not provide information on the optimal dose for individuals with obesity. The authors used the Preferred Items for Systematic Reviews and Meta-Analysis guidelines with multiple databases, Google Scholar, and English language articles to systematically assess the vancomycin-response relationship. Based on their findings, the authors concluded that the initial vancomycin dose referenced to the total body weight could be a better predictor of vancomycin trough concentration rather than adjusted or actual body weight. This review is meaningful as it highlights the methods for vancomycin dosage prediction among patients with obesity. However, the dataset and sample size were small, which may have influenced the results. Furthermore, the clinical outcomes, nephrotoxicity, and other adverse effects associated with high vancomycin doses were not assessed. Therefore, further studies are warranted to reach a relevant conclusion regarding the vancomycin dosage approach in overweight or obese individuals infected with bacteria.

Baral and Koh Jing Jie et al. introduce bacteriophages and dual-acting antibiotic hybrids as the viable replacement for antibiotics in the future. Bacteriophages, viruses that infect bacteria, were first used in the clinic in 1919 but were overshadowed by the discovery of antibiotics and now have a renaissance in industry, medicine, food processing, and biotechnology (Roy et al., 2018; Bao et al., 2019; Stachler et al., 2021). Baral introduced the benefits of phage therapy, including specificity, increased local antibacterial effects, and limited AMR growth and spread. Furthermore, bacteriophages are used as supplements to antibiotics (Torres-Barceló and Hochberg, 2016), especially in eradicating biofilm-forming bacteria (Roy et al., 2018). However, drawbacks of phage therapy for practical use in medicine have been highlighted. These include determination of the precise type of infectious bacteria to use appropriate phages, non-ideality for systemic diseases, limited availability, and appropriate regulation.

As a promising strategy to treat AMR, hybrid antibiotics, which are single hybrid molecules of two or more pharmacophores of antibiotics with dissimilar modes of action against target bacteria, were introduced by Kho Jing Jie et al. (2022). This strategy is beneficial in terms of better antibacterial activity and overcoming resistance to individual antibiotics by targeting AMR bacteria and simultaneously inhibiting or killing the bacteria. However, this strategy has two limitations that may affect the overall yield and intrinsic activity: 1) the complexity in designing chemical synthesis (Ma and Lynch, 2016; Domalaon et al., 2018; Lungu et al., 2022) and 2) drug permeability impediments in Gram-negative bacteria. This can be remedied by conjugation with non-antibacterial synergistic adjuvants, such as small molecules or biologics, by modifying anti-resistance mechanisms, such as bypassing membrane barriers or immune cell stimulation (Kho Jing Jie et al., 2022). Examples include adjuvants conjugated with antibiotics, such as Fetroja^®^, which bypass the outer membrane of Gram-negative bacteria via the iron-uptake pathway (Syed, 2021). Therefore, hybrid antibiotics can be a viable next-generation approach to expand the antimicrobial arsenal; however, molecular modeling studies and computational strategies are required for designing antibiotic hybrids for early-stage development.

Finally, Makarem et al. introduced the benefits of low-molecular-weight heparin (LMWH; Lovenox) for COVID-19 therapy. LMWH was introduced to clinics in 1935 and is now widely used as an anticoagulant in the treatment of venous thromboembolism, cardiovascular disorders, stroke, and thrombosis prophylaxis (Qiu et al., 2021). Recently, proper management of hypercoagulation using LMWH in COVID-19 patients has been associated with reduced mortality (Ayerbe et al., 2020; Di Castelnuovo et al., 2021). Despite the potential therapeutic advantages of LMWH treatment, there still remains several concerns. Therefore, more multicenter, placebo-controlled, high-quality, randomized clinical trials with plainly outlined baseline characteristics and outcomes are urgently needed to evaluate the efficacy of LMWH in COVID-19 therapy in clinical practice.
